# FRED: Exergame to Prevent Dependence and Functional Deterioration Associated with Ageing. A Pilot Three-Week Randomized Controlled Clinical Trial

**DOI:** 10.3390/ijerph14121439

**Published:** 2017-11-23

**Authors:** Iranzu Mugueta-Aguinaga, Begonya Garcia-Zapirain

**Affiliations:** 1 Rehabilitation Service, Cruces University Hospital, Plaza Cruces s/n, 48903 Barakaldo, Spain; 2 eVIDA Lab, University of Deusto, Avda. Universidades, 24, 48007 Bilbao, Spain; mbgarciazapi@deusto.es

**Keywords:** frailty, elderly people, exergame, physical activity, kinect

## Abstract

*Introduction:* Frailty syndrome and advanced age may decrease the acceptance of illness and quality of life, and worsen patients’ existing health conditions, as well as leading to an increase in health care expenses. *Purpose:* The purpose of this study is to reduce frailty risk via the use of a FRED game which has been expressly designed and put together for the study. *Materials and Methods:* A total of 40 frail volunteers with a score of <10 points in the short physical performance battery (SPPB) took part in a feasibility study in order to validate the FRED game. Following randomisation, the study group (20 subjects) took part in nine sessions of 20 min each over a three-week period. The control group (19 subjects) continued to lead their daily lives in the course of which they had no physical activity scheduled; *Results:* After three weeks and having taken part in nine physical activity sessions with the FRED game, 60% of subjects from the study group (12/20) obtained a score of ≥10 points at the end of the study, i.e., less risk of evidencing frailty. This result proved to be statistically significant (*p* < 0.001). The degree of compliance with and adherence to the game was confirmed by 100% attendance of the sessions. *Discussion:* Our findings support the hypothesis that FRED, an ad hoc designed exergame, significantly reduced the presence and severity of frailty in a sample of sedentary elders, thus potentially modifying their risk profile. *Conclusions:* The FRED game is a tool that shows a 99% certain improvement in the degree of frailty in frail elderly subjects. The effectiveness of the design of ad hoc games in a certain pathology or population group is therefore evidenced.

## 1. Introduction

A recent international consensus defines physical frailty as a major “medical syndrome with many causes and contributing factors that is characterised by a reduction in strength, resistance and physiological function that increases individual vulnerability leading to the development of greater dependence and/or death” [1].

Frailty is an important predictor of adverse outcomes in elderly people, such as death (up to 45% a year), institutionalisation, falls, mobility decline, increased disability in basic and instrumental activities of daily living (BADL/IADL) and hospitalisation, and those with pre-frailty have an increased risk of becoming frail within 3 years [2].

Institutionalised older adults are a heterogeneous population in terms of disability rates, multi-morbidity, quality of life and vulnerability. Interventions in this population should be individualised, and the detection and treatment of frailty could be of use in preventing disability, mobility decline, falls and mortality [2].

Frailty is a state of extreme vulnerability to endogenous and exogenous stressors that expose the individual to a higher risk of negative health-related outcomes. It may represent a transitional phase between successful ageing and disability, and a condition to target for restoring robustness in the individual at risk. Given its syndromic nature, targeting frailty requires a comprehensive approach. Identifying frailty as a target in order to implement preventive interventions against age-related conditions is pivotal, and so every effort should be made by healthcare authorities to maximise efforts in this field by balancing priorities, needs, and resources. Raising awareness about frailty and age-related conditions in the population is important for effective prevention, and should lead to the promotion of lifelong healthy behaviour and lifestyles [3].

By observing the major role played by technology in our modern-day society, we may ask the following question: to what extent does technology relate to frailty or, put another way, which technological resources are used to deal with frailty? To respond to this question, we decided to conduct a review in order to ascertain the state of the art from January 2005 to December 2015. The following conclusions were drawn from this state of the art:

- The application of technological solutions in health care is a field that is constantly expanding and about which there are great expectations both on the part of users and healthcare professionals and carers, despite the reticence existing in this area known as a technology gap [4].
- It is of the utmost importance to continue working to reduce the gap existing between technology, frail elderly persons, healthcare professionals and carers by bringing together the different views about technology and thus stimulate dialogue, an increased awareness and knowledge about the respective fields in order to engage in collaboration on projects that may reduce costs and improve health and quality of life [4].

The last decade has seen a rapid increase in research into the use of technology in the elderly population [5,6]. Exercise via interactive video games—known as exergames—is being increasingly used to increase physical activity, thus improving health and the physical function in elderly persons [5,7,8,9].

The concept of the exergame may be defined as the use of a video game that incorporates physical activity into the game, leading to an improvement in motivation and adherence to participation in that physical activity [10].

Exergames may have basic advantages over traditional exercise, as they easily enable specific tasks to be performed via a range of difficulty levels. This in turn enables the user to start at a suitably challenging level and then to gradually progress in terms of level of difficulty, based on individual performance in real time [5]. However, commercial games that are easily available are mainly designed for entertainment and recreational purposes for younger users with more complex interaction and interface. Exergaming technology is therefore now less of a feasible option for many elderly people [5,11,12] and, furthermore, games available on the market are mainly designed for enjoyment and are not based on basic exercising principles. For games to be effective, users need to make movements with specific features during the game that may be deemed relevant for the function being trained.

As a physiotherapist, the first author of this article designed the contents of the game to help elderly persons improve their physical capacity and their state of health and independence in the activities they carry out in their daily life via the game in which they take part. The aim is therefore to reduce frailty risk or, if frailty already existed, to improve the extent to which it is in evidence.

In addition to overcoming the technological barrier existing between elderly persons and the use of technological devices, the aim is also to ensure that the game has extra appeal over those already existing such as Wii sport (bowling, tennis, boxing) and Wii fit [13,14,15], in which the scenarios and movements are unique. The extra appeal offered by FRED is that the user passes through different scenarios in which they carry out a range of activities with a specific objective in mind or within a main activity.

The ultimate goal is for the users to be the protagonist, for them to like the game, and for them to find it appealing so as to become more involved in the exercise and pursue it further [16].

An attempt is made to validate the FRED game via the feasibility pilot study that is described below.

## 2. Materials and Methods

### 2.1. Participants

Approval was requested and obtained from the Ethics Committee in Research of the Deusto Foundation (University of Deusto), reference number Ref: ETK-17/15-16, in order to proceed with the study. Contact was established with two residential homes, and the necessary permits were requested and obtained from the management at the home. Recruitment at both residential homes was undertaken via informative talks that were given. These talks were open to all interested residents and were advertised via informative posters and pamphlets.

Inclusion criteria: persons over 65 years of age with a Barthel score equal to or above 90 points who carry out no scheduled physical activity.

Exclusion criteria: persons over 65 years of age with a Barthel score less than 90 points or with a Barthel score equal to or above 90 points who carry out scheduled physical activity.

**Figure 1:**
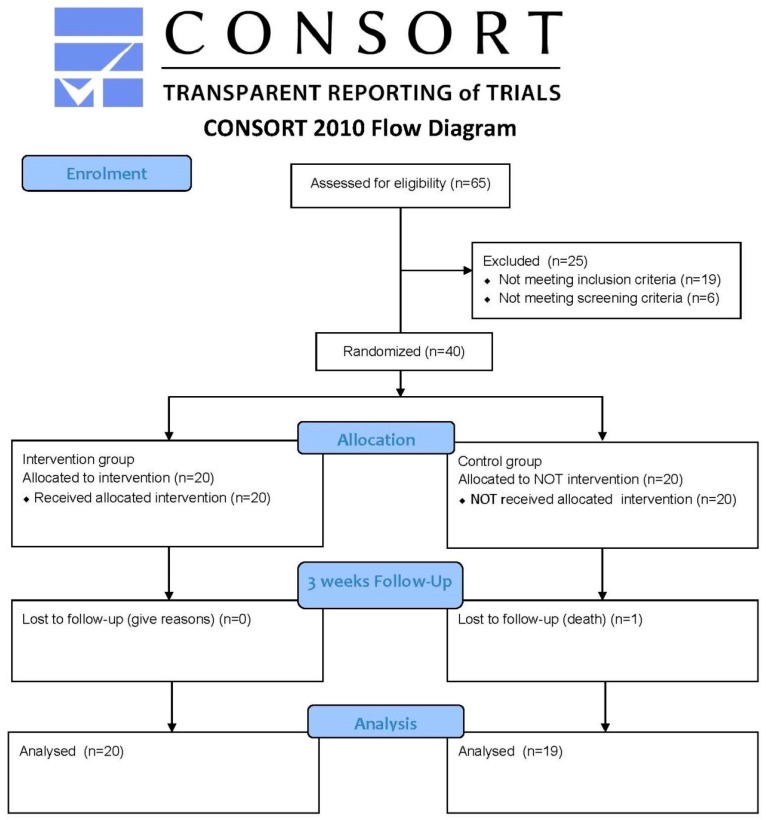
CONSORT flow diagram of the progress through the phases of a parallel randomised trial of two groups (i.e., enrolment, intervention allocation, follow-up, and data analysis). Source: [19,20].

46 individuals met the inclusion criteria, and these subjects were again asked to carry out some specific tests in order to evidence their degree of frailty (Figure 1).

Barthel Index is a scale in which a quantitative estimate is obtained of the degree of independence a subject has to pursue activities of daily living. The score ranges 0–100 [17,18], in which value 0 means total dependence and value 100 means independence.

### 2.2. Experimental Design

The possibility was considered of carrying out a feasibility study via the design of a pilot three-week randomized controlled clinical trial (Phase 1).

The purpose of the experiment was to reduce the risk of frailty in the subjects.

#### 2.2.1. Evaluation Test

Frailty screening was undertaken using the short physical performance battery (SPPB), which was validated and normalised within our milieu and combines balance testing, gait speed and chair stand. This prioritization was based on its successful validation in detecting frailty and great reliability in predicting disability [21,22].

There is considerable evidence to be found in international literature that shows the close association existing between the SPPB and many functional state measures [23].

**Figure 2:**
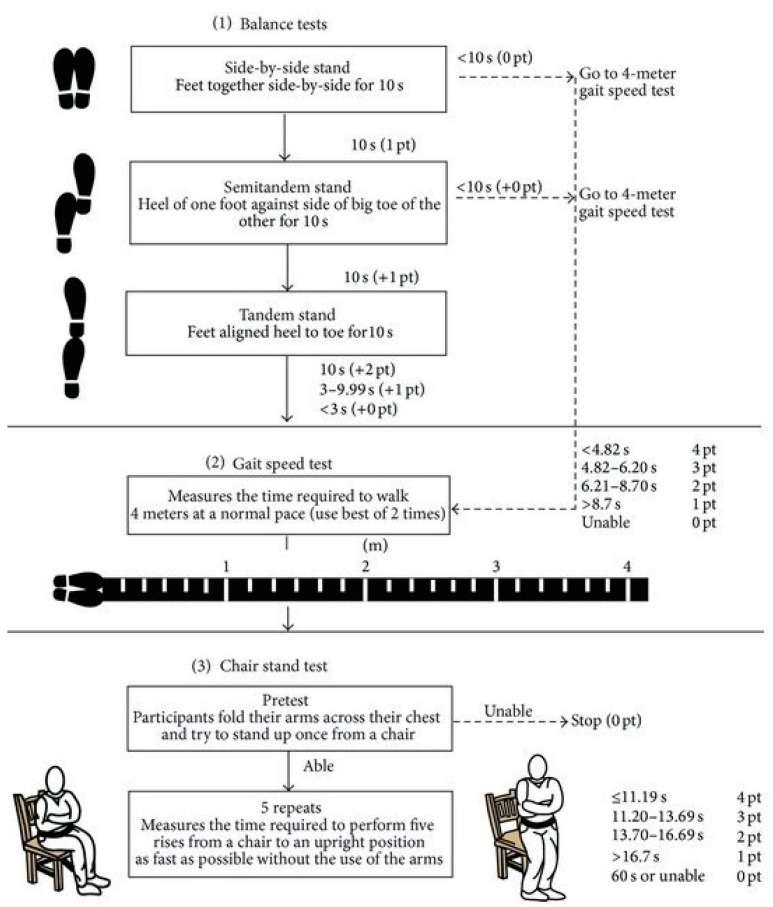
Short physical performance battery (SPPB) flowchart. Source: [27].

The short physical performance battery (SPPB) is a group of measures that combines the results of the gait speed, chair stand and balance tests. These tests follow a hierarchical sequence (Figure 2).

It has been used as a predictive tool for possible disability and can aid in the monitoring of function in older people. The scores range from 0 (worst performance) to 12 (best performance). Changes of one point in the SPPB result have clinical significance [24,25].

The SPPB has been shown to have predictive validity showing a gradient of risk for falls, mortality, nursing home admission (dependency status), and disability, i.e., frailty risk.

A score lower than 10 points indicates a high risk of disability and also dependency, falls and even death, i.e., frailty risk [26].

Subjects who achieved the inclusion criteria (46) were cited to perform frailty screening tests with the Short Physical Performance Battery (SPPB). Of the 46 subjects, 40 obtained scores below 10, i.e., 40 subjects evidence frailty risk according to the SPPB test.

**Figure 3:**
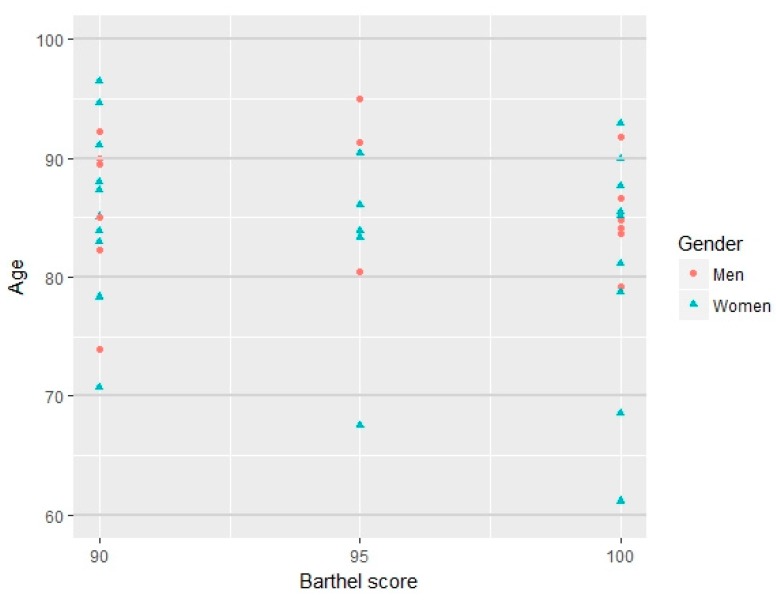
Presentation of participants who meet inclusion criteria according to ranges of age, gender and Barthel score.

After frailty screening, to undertake randomization, the 40 subjects were classified according to age, sex, and Barthel Index (Figure 3), resulting in a study group (20) and a control group (20) of subjects respectively.

The subjects belonging to the study group carried out three sessions per week over a three-week period. Each session involved 20 min of activity divided into three phases. The first targeted both the upper and lower extremities, while the second and third targeted the specifically the upper and lower extremities respectively. The study group was supervised while doing the FRED game by a monitor.

The subjects belonging to the control group continued with their daily life.

After three weeks had elapsed and the subjects had carried out nine physical activity sessions using the FRED game, the short physical performance battery (SPPB) was repeated to check whether the degree of frailty had been reduced.

#### 2.2.2. The Game

The FRED game was designed as a type of exergame [10], in which the first author of this article, who is a physiotherapist, designed the contents of the game while at the same time taking into account both specific movements for developing physical exercise and the devising of different scenarios in which these movements could be developed [28,29]. These scenarios are developed in a logical order to ensure that the individuals who perform them find a meaning to the activity. Each movement is designed by taking into account both biomechanical and neuromotor parameters and evidence features of sufficient extent to be recognised by the Kinect sensor [30].

FRED is a game that has been developed using a 3D unity motor, and needs a Kinect game controller connected to a computer and a screen or TV.

**Description of the FRED game:** The game consists of various scenarios. Each scenario represents one or more steps in a simplified process to produce txakoli [28,31]. The user starts to carry out the different activities which, as well as being remote controlled and in order, correspond to a specific movement of the upper and/or lower extremity. Apart from the physical activity, the game requires attention, coordination of movement, balance, accuracy and spatial orientation.

The Kinect sensor detects and records the time and ranges of movement, which are both previously defined. A positive score is given for successfully-completed exercises the final score is shown at the end of each phase [30].

The movements made in the game FRED are not based on degree but more on being a functional movement. For this reason, the basic parameters were given to the software architect for each movement to get the objective in each scenario. After that, the software architect built the algorithm. Finally, the movement was tested to verify that the goal was reached in each scenario.

The algorithm is built taking in to account the different segments and points that correspond to different joints, muscles and bones.

Kinect does a reading of what is in front of the camera and recognizes the body of who is playing. Once this is done, it divides the body parts into joints, that is, connecting points related to the body joints to generate a skeleton.

**Figure 4:**
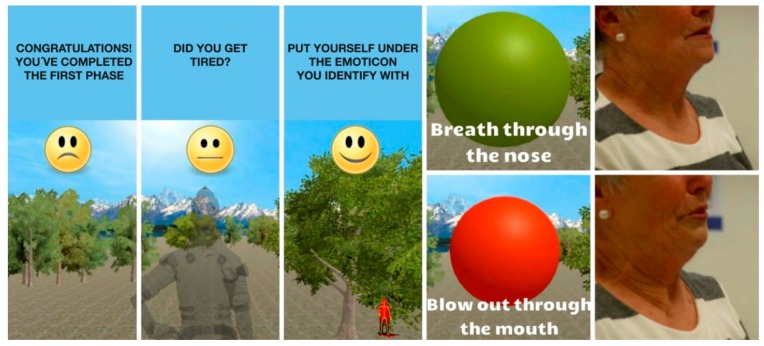
Screen images for when effort is rated, together with screen images for the breathing exercises.

Below is a description of the game scenarios that have been developed for the feasibility study. These scenarios are distributed into three phases with a total 20 min duration. On completion of each phase, the player has the chance to rate the effort made using the simplified Borg scale [32]. Depending on their rating, they will either be able to continue immediately, or after doing some abdominal-diaphragmatic exercises for a period of time that depends on the effort rating. Otherwise, they may abandon the activity (Figure 4).

In sequential order, we have:

• Part 1

**Scenario 1:** In which the player walks on a path towards a farmhouse along which they have to avoid different obstacles: stones, streams (by crossing bridges, tree trunks and branches).

**Figure 5:**
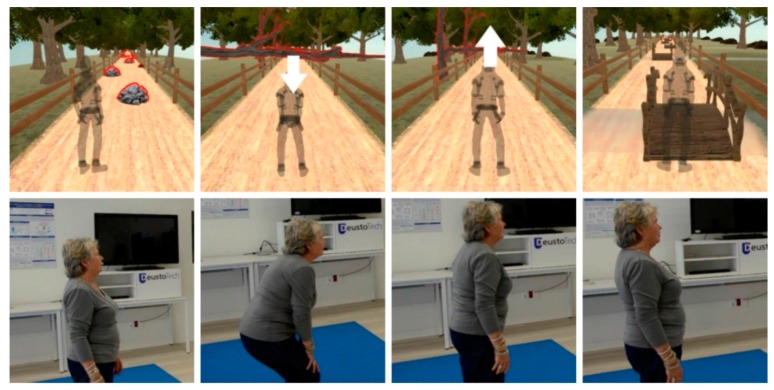
Images of the game in Scenario 1.

The specific movements to be made in Scenario 1 are lateral movements; bending down and getting up (flexion-extension of trunk, hip, knees and ankles); alternate lifting of the legs while supporting bodyweight and maintaining balance; alternate lifting of the arms (Figure 5).

These specific movements also require attention, coordination of movement, balance, accuracy and spatial orientation.

**Scenario 2:** At the end of the path, while covering the last few metres before reaching the farmhouse, the player has to avoid an obstacle while at the same time picking an apple. These movements will be alternately shown in homolateral and contralateral form.

**Figure 6:**
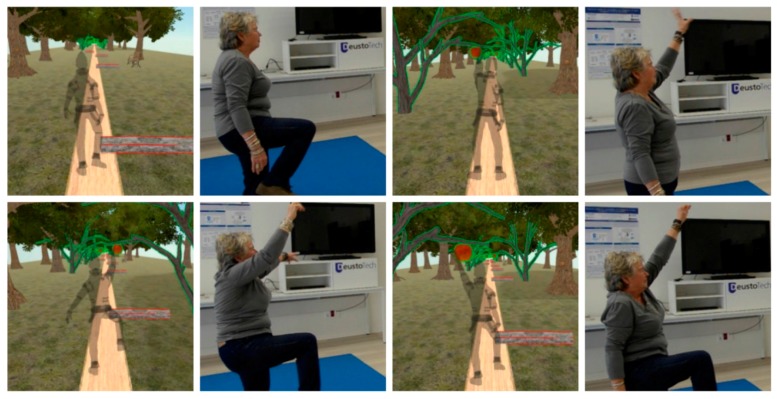
Images of the game in Scenario 2.

The specific movements to be made in Scenario 2 are supporting bodyweight and maintaining balance together with homolateral lifting of arm and leg; supporting bodyweight and maintaining balance together with contralateral lifting of arm and leg (Figure 6).

These specific movements also require attention, coordination of movement, balance, accuracy and spatial orientation. 

• Part 2

**Scenario 3:** The player reaches the farmhouse, picks up a hose outside and unrolls it.

**Figure 7:**
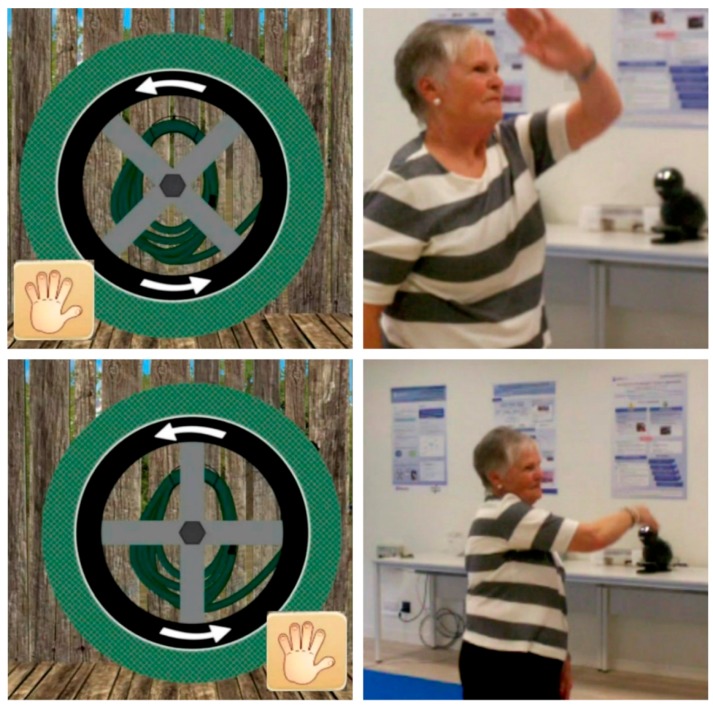
Images of the game in Scenario 3.

The specific movement to be made in Scenario 3 is the circumduction movement of the shoulder on the frontal plane. The movement is made with both the left and right shoulder (Figure 7).

These specific movements also require attention, coordination of movement, balance, accuracy and spatial orientation.

**Scenario 4:** Watering the vines with the hose. The user observes how the vines grow while they are watering them.

**Figure 8:**
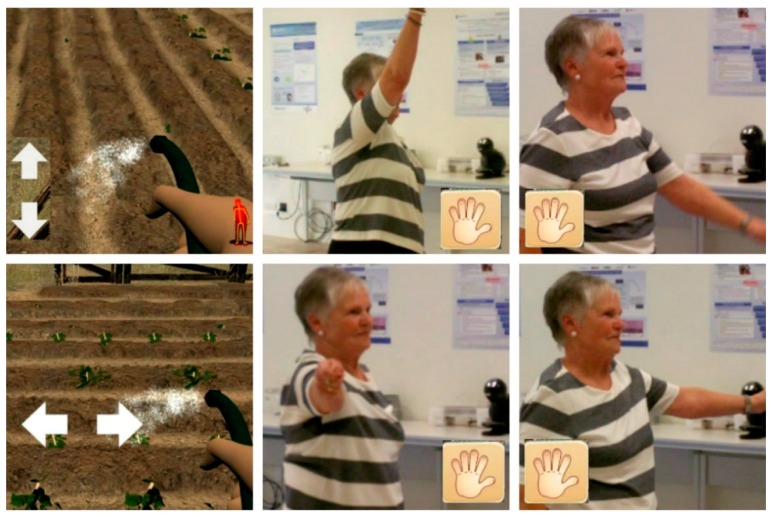
Images of the game in Scenario 4.

The specific movements to be made in Scenario 4 are the flexion-extension movements and abduction/adduction to different extents <90°. Movements are made both with the right and left hand (Figure 8).

These specific movements also require attention, coordination of movement, balance, accuracy and spatial orientation.

**Scenario 5:** The player rolls up the hose.

The specific movement to be made in Scenario 5 is the circumduction of the shoulder on the frontal plane. The movement is made both with the left and right shoulder (Figure 7: in the opposite direction both for the left and right arms).

These specific movements also require attention, coordination of movement, balance, accuracy and spatial orientation.

**Scenario 6:** The player picks grapes and puts them in a bucket.

**Figure 9:**
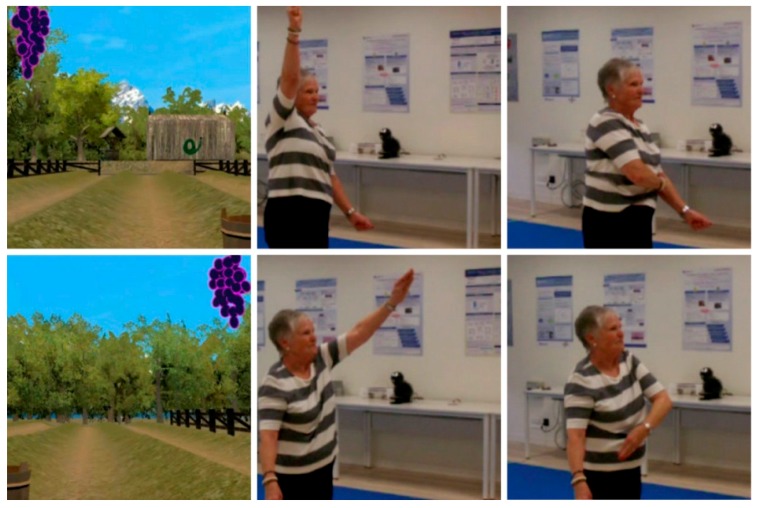
Images of the game in Scenario 6.

The specific movements to be made in Scenario 6 are flexion-extension movements, lifting the shoulder >90°; internal and external rotation of the shoulder. Movements are made with both the left and right hand (Figure 9).

These specific movements also require attention, coordination of movement, balance, accuracy and spatial orientation. 

• Part 3

**Figure 10:**
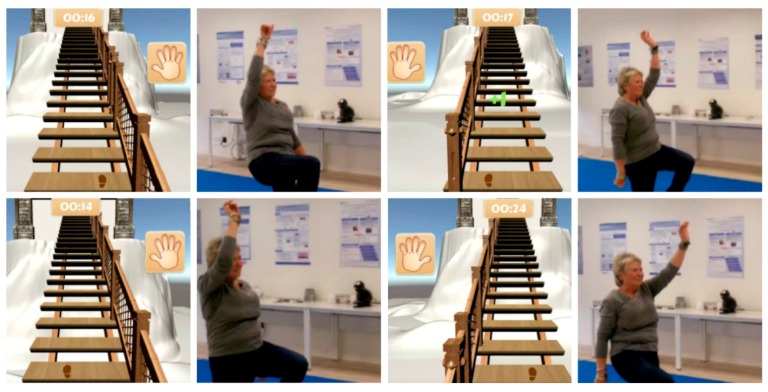
Images from the game in Scenario 7.

**Scenario 7:** The player climbs the stairs inside the house, grabbing the railings. This is done with a homolateral movement (arm and leg on the same side) and with a contralateral movement (arm and leg on opposite sides) (Figure 10).

These specific movements also require attention, coordination of movement, balance, accuracy and spatial orientation. 

### 2.3. Statistical Analysis

The R open code statistical programme version 3.2 (The R Foundation for Statistical Computing, Vienna, Austria) for Windows is used to carry out the statistical tests and create the graphs.

The Wilcoxon Exact Test is carried out in order to compare the means obtained from the study group before the test and after three weeks. 

The relationship between an improvement in results and being in the study group was measured using the Pearson and Fisher-Exact tests.

An adjusted Cochran-Mantel-Haenszel estimate was used to compare the relative risks involved when stratifying the data in terms of age and gender.

## 3. Results

### 3.1. Description of the Sample in Week 1

40 subjects started the study, although only 39 completed it. One subject from the control group passed away before the study was completed.

The sample proved to be homogeneous in terms of the Barthel Index, age, gender and frailty risk according to the SPPB score obtained (Table 1).

**Figure 11:**
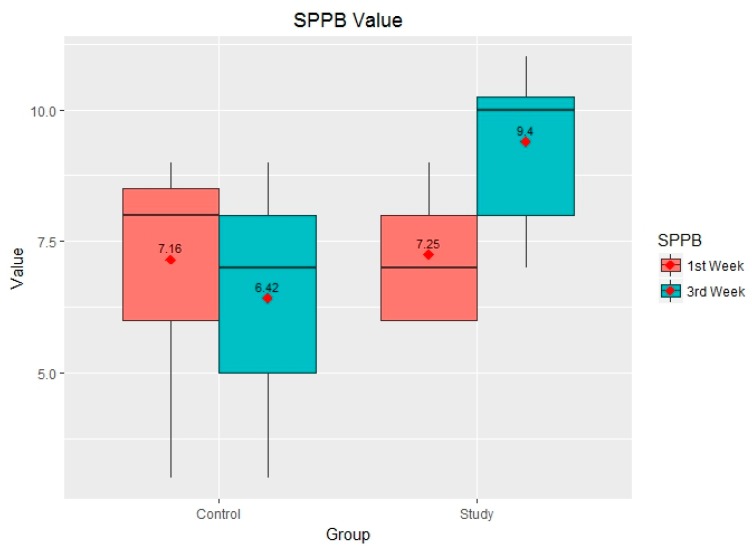
Statistical description of the short physical performance battery (SPPB) results in the first and third weeks of the study.

In the first week of the study it was noted that both groups were at 100% risk of evidencing frailty and it could also be observed that although the control group evidence a greater minimum and maximum range than the study group, both means were similar. The boxes and whiskers represent the minimum, the first quartile, the median, the third quartile and the maximum, both for the control group and for the study group in the 1st and 3rd week (Figure 11).

### 3.2. SPPB after Three Weeks

**Figure 12:**
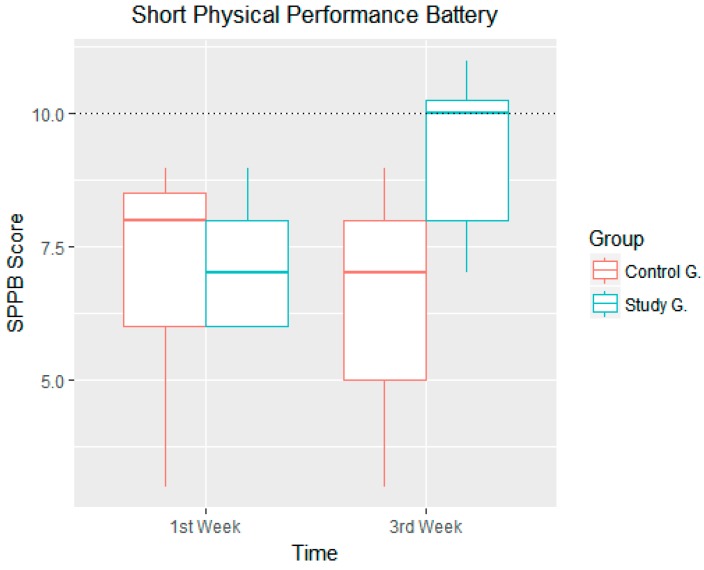
Score obtained using the SPPB in weeks 1 and 3.

After analysing the results obtained in the tests carried out using the SPPB, after three weeks and after having conducted nine physical activity sessions using the FRED game, it was noted that the results from the study group increased whereas those from the control group decreased, as can be seen in Figure 11. On the one hand, 25% of patients from the control group obtained scores between 5 and 7, whereas at the start of the study (week 1) they obtained scores between 6 and 8. Conversely, we also note a substantial improvement in scores obtained by patients from the study group—the minimum increase was from 6 to 7, and 50% of patients obtained scores of 10 or over, which means that these patients are no longer considered to be exposed to frailty risk according to the SPPB tests (Figure 11 and Figure 12).

The Wilcoxon test was carried out to determine that the results from the study group improved after three weeks. This test enabled the initial hypothesis to be rejected that the results obtained by the study group before and after would be the same, with a value of *p* < 0.001.

Figure 11 shows the evolution of results obtained from both groups, broken down using a bar chart that shows whether subjects improved or otherwise, and whether subjects ended up exceeding the frailty limit (SPPB ≥ 10).

Dependence between an improvement in results and belonging to the study group was measured via Pearson and Fisher-Exact tests. These tests show that the SPPB results depend on the group to which the subject belongs, with a value of *p* < 0.001.

**Figure 13:**
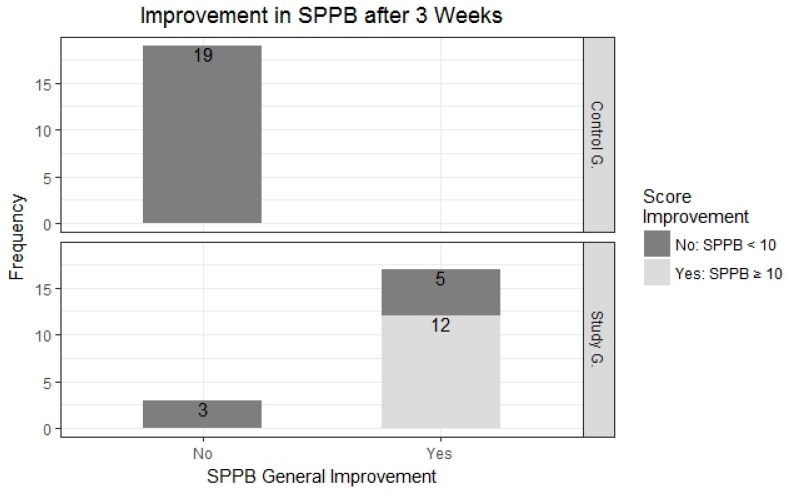
Frailty percentage and number of subjects in control group and study group at the end of week 3.

By the third week, we noted that all 19 subjects from the control group (100%) evidenced frailty risk, whereas the 20 subjects from the study group only evidenced a frailty risk rate of 40%. The results confirmed the fact that 60% of subjects from the study group (12 of the 20 patients) no longer evidenced frailty risk after the third week. Within the general framework, 85% (17 of the 20 patients) of subjects from the study group showed some type of improvement in terms of their SPPB results (Figure 13).

**Figure 14:**
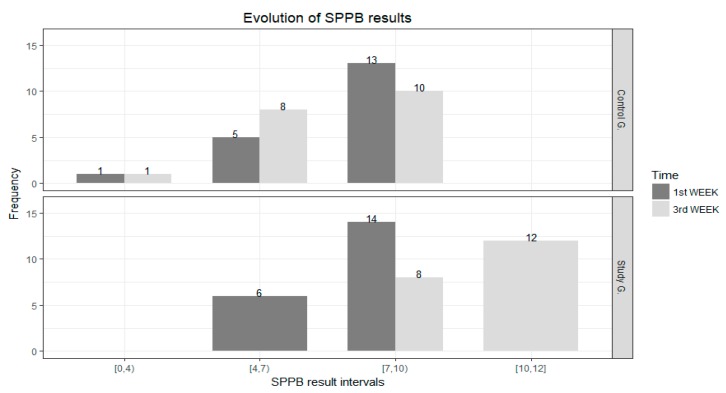
SPPB score evolution.

Below is a description of the SPPB scores of the subjects from the tests conducted in the first week and after three weeks, according to SPPB result intervals (Figure 14).

Considering the evolution of SPPB results and the marked intervals, we note that three subjects from the control group dropped from the interval (7, 10) to the one immediately below, whereas all the subjects from the interval (4, 6) in the study group moved up to higher intervals (three subjects to the interval (7, 10) and three subjects to the interval (10, 12)). Additionally, nine subjects from the interval (7, 10) moved up to the interval (10,12).

It was not necessary to compare SPPB results with regard to age or gender as the effect of these variables was minimised by having a homogenous sample of subjects in the control and study groups.

**Figure 15:**
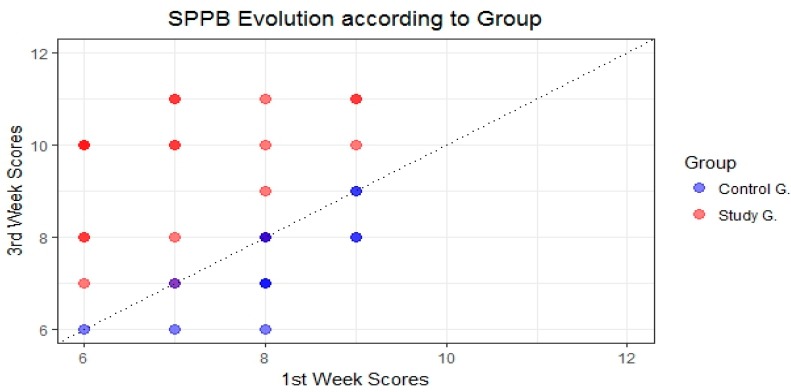
Distribution of SPPB score obtained according to age and gender.

After three weeks, all control group subjects’ relative risk of obtaining an SPPB result below 10 is 2.5 times greater than that for all of the study group subjects (Figure 15). The relative risk according to age of the control group (Cochran-Mantel-Haenszel estimate) for two strata (<85 and ≥85 years) is 2.53 greater than for the study group under the same conditions, while the relative risk according to gender of the control group is 2.52 times greater than for the study group, also under the same conditions.

It was noted that the relative risk of all subjects from the control group in comparison to the study group was nearly identical to the relative risks according to age and gender, whereby these variables were not considered distortion factors (these are known as confusion variables in statistics). This was due to the homogenous nature evidenced by the control and study groups in terms of age and gender.

It was not necessary to compare SPPB results with regard to age or gender as the effect of these variables was minimised by the fact of having a homogenous sample of subjects in the control and study groups.

### 3.3. Game Satisfaction

After completing the game each day, each participant was asked two questions with the possible responses being YES or NO:

1. Do you like the game?
2. Do you find it motivating for the purpose of improving your physical condition?

**Figure 16:**
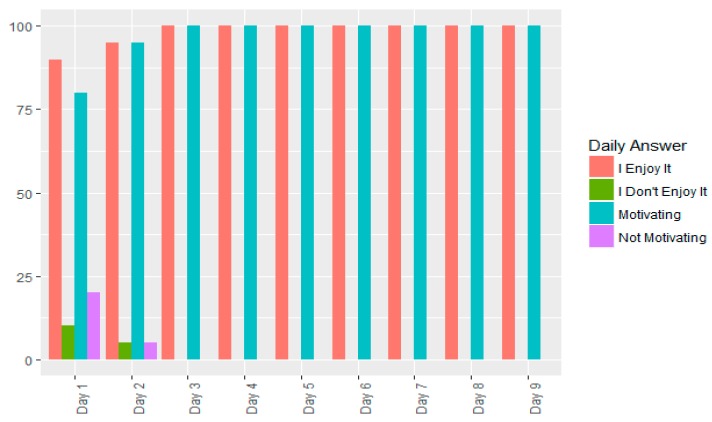
Daily response percentages by the study group to the questions: “Do you like the game?” and “Do you find it motivating for the purpose of improving your physical condition?”

As regards the first question, except on days 1 and 2 when, respectively 10% (2 subjects) and 5% (1 subject) of subjects gave a negative response, the 20 subjects from the study group responded YES on the other days (Figure 16).

As regards the second question, except on days 1 and 2 when, respectively, 20% (4 subjects) and 5% (1 subject) of subjects gave a negative response, the 20 subjects from the study group responded YES on the other days (Figure 16).

## 4. Discussion

The results obtained are discussed in this section by contrasting them with those of other authors who have worked on related themes.

Our findings support the hypothesis that FRED, an ad hoc designed exergame, significantly reduced the presence and severity of frailty risk in a sample of sedentary elders [33], thus potentially modifying their risk profile.

Our results, consistent with previous evidence, suggest that elders with higher risk profiles may still benefit from preventive strategies and should not a priori be excluded from interventions to prevent disability.

Cesari el al. [34] conducted a study in which the physical activity programme included aerobics (walking), strength, flexibility and balance training which were undertaken in three phases over a 12-month period: adoption (weeks 1–8), transition (weeks 9–24) and maintenance (week 25 to month 12). The results suggested that regular physical activity may reduce frailty, especially in individuals at greater risk of disability. Future studies should be aimed at testing the possible benefits produced by multi-domain interventions in frailty.

Giné-Garriga et al. [35] conducted a study with a combination of functional balance and lower-body, strength-based exercises over a 12-week period, referred to as the functional circuit training program (FCT). This involved setting aside one day per week for balance training and another day for lower-body strength-based exercises lasting 15 min each. The results suggested that the functional circuit training program (FCT) is effective in improving self-reported measures of fear of falling and health status in a group of physically frail individuals.

Clegg et al. [36] compared the effectiveness of the HOPE (Home-based Older People’s Exercise) programme with usual care via a blind pilot randomised controlled trial (RCT) over a 12-month period, completing 15 min of the programme three times per day, five days per week. The results suggested that the HOPE programme trial provided preliminary evidence that the deterioration in mobility experienced by older people with frailty may be reduced through a 12 week exercise intervention.

The above studies [34,35,36] combined different types of physical activity and were performed over longer periods of time and at greater weekly frequency than the physical activity in our study using the FRED game. Although they manage to reduce frailty, they require far more time to do so, and none of them considers exercising by using an exergame. Therefore, the degree of frailty can be reduced in less time using the FRED game because after using the FRED game during only 3 weeks the results confirmed the fact that 60% of subjects from the study group (12 of the 20 patients) no longer evidenced frailty risk when the third week was over. Within the general framework, 85% (17 of the 20 patients) of subjects from the study group showed some type of improvement.

The study conducted by Daniel et al. [14] uses the exergame as a physical activity to be carried out in order to reduce frailty. In this case, they use the Nintendo^®^ Wii™ console with general games designed for all. These include bowling, tennis and boxing. They then compare them to seated exercise and a control group. They carried out 45 min exercise sessions three times per week for 15 weeks. The results suggested that all the differences reflected improved physical functional status in the seated exercise or Wii-fit groups compared to the control group. Wii games are varied and interactive—some games are individual (downhill skiing or skateboarding) and other games are interactive, involving multiple players (tennis, bowling or boxing). They could therefore offer home-based participants greater variety (and potentially increased engagement) than a standard seated exercise protocol. The FRED game differs from the above study because it managed to reduce the degree of frailty in a fifth of the time (three weeks as opposed to 15), including sessions of less than half the duration (20 min as opposed to 45 min). Consequently, the degree of frailty can be reduced in less time using the FRED game.

The study conducted by Van Diest et al. [13] used the exergame for unsupervised balance training at home—a virtual ice skater for 30 min, three times per week for six weeks. The pilot study showed that unsupervised home-based exergaming is feasible in community-dwelling older adults, but also that participants do not benefit equally from the programme, thereby emphasizing the need for more personalized exergame training programmes. This conclusion without doubt highlights the ad hoc feature of FRED in terms of the design and compilation of exercises.

Bieryla KA [37] used the Xbox Kinect™ to improve balance in elderly people. The results showed an improvement in terms of the balance tests carried out. However, no improvements were evidenced in the Timed Up and Go (TUG) test, which the author did not expect given that use of the Kinect™ compels participants to move more than other game systems.

Scheer et al. [38] assessed the effects of using Nintendo Wii, Sony Move and Microsoft Kinect™ on heart rate, ventilation, oxygen consumption and energy expenditure when competing against a computer or human opponent in young adults. The resulting data for oxygen consumption was used to compare the Nintendo Wii, Sony Move and Microsoft Kinect™ games using moderate health guidelines to promote physical activity.

O’Donovan et al. [39] considered comparing energy expenditure when playing in individual and multi-player mode using Xbox Kinect™ and Wii™ consoles. They concluded that energy expenditure and heart rate increased when playing with the Xbox Kinect™ console in multi-player mode.

Holmes et al. [40] sought to determine training exercise intensity using the Xbox Kinect™ in adults over the age of 18 who had been diagnosed as having cystic fibrosis (CF), an illness in which physical exercise forms a very important part of therapy. They concluded that training with the Xbox Kinect ™ represents a high-intensity exercise for adults with CF and may be considered a suitable alternative to conventional types of exercise.

## 5. Conclusions

After carrying out the analysis, the conclusion can be drawn that the FRED game has been shown to have a major impact on the short physical performance battery (SPPB) results obtained by subjects. The improvement in SPPB results means that the risk of evidencing frailty was reduced after nine sessions (three weeks of 20 minutes physical activity performed three times a week).

Furthermore, it was shown to have a major motivating impact owing to the fact that 100% compliance was obtained, because the 20 subjects took part in all the sessions. Adherence to the game was confirmed because 100% of subjects responded affirmatively to the relevant questions from the third until the ninth session.

In view of the excellent results, we are considering carrying out a two- to six-week phase pilot study, including the Barthel questionnaire, physiological variables (biofeedback), a questionnaire about state of health and a further questionnaire about the use of software.

## Figures and Tables

**Table 1:** Description of sample features.

	Control Group (*n* = 19)	Study Group (*n* = 20)
Barthel	Slightly dependent 60% Independent 40%	Slightly dependent 60% Independent 40%
Age	83.11 ± 9.01 years	85.47 ± 6.46 years
Gender	Men 40% Women 60%	Men 40% Women 60%
Frailty RISK	100%	100%
Sppb	7.16 ± 1.07	7.25 ± 1.86
